# Клинические рекомендации «Ожирение у детей»

**DOI:** 10.14341/probl12802

**Published:** 2021-08-20

**Authors:** В. А. Петеркова, О. Б. Безлепкина, Н. В. Болотова, Е. А. Богова, О. В. Васюкова, Я. В. Гирш, А. В. Кияев, И. Б. Кострова, О. А. Малиевский, Е. Г. Михайлова, П. Л. Окороков, Е. Е. Петряйкина, Т. Е. Таранушенко, Е. Б. Храмова

**Affiliations:** Национальный медицинский исследовательский центр эндокринологии; Национальный медицинский исследовательский центр эндокринологии; Саратовский государственный медицинский университет им. В.И. Разумовского; Национальный медицинский исследовательский центр эндокринологии; Национальный медицинский исследовательский центр эндокринологии; Сургутский государственный университет ХМАО-Югры; Уральский государственный медицинский университет; Детская республиканская клиническая больница им. Н.М. Кураева; Башкирский государственный медицинский университет; Детская городская клиническая больница №1 им. Н.Н. Ивановой; Национальный медицинский исследовательский центр эндокринологии; Российская детская клиническая больница; Красноярский государственный медицинский университет имени профессора В.Ф. Войно-Ясенецкого; Тюменский государственный медицинский университет

**Keywords:** клинические рекомендации, ожирение, дети

## Abstract

Ожирение у детей является актуальной проблемой детской эндокринологии в связи с широкой распространенностью, развитием метаболических нарушений и их устойчивым трекингом во взрослую жизнь. Разработанные клинические рекомендации являются основным рабочим инструментом практикующего врача. В них кратко и структурированно изложены основные сведения об эпидемиологии и современной классификации ожирения, методах его диагностики и лечения, базирующихся на принципах доказательной медицины.

## Abstract (en)

Childhood obesity is an urgent problem of pediatric endocrinology due to the widespread occurrence, the development of metabolic complications and their steady tracking into adulthood. The developed clinical guidelines are the main working tool of the practitioner. They briefly and structurally present the main information about the epidemiology and modern classification of obesity, methods of its diagnosis and treatment based on the principles of evidence-based medicine.

СПИСОК СОКРАЩЕНИЙ

ТЕРМИНЫ И ОПРЕДЕЛЕНИЯ

## 1. КРАТКАЯ ИНФОРМАЦИЯ ПО ЗАБОЛЕВАНИЮ ИЛИ СОСТОЯНИЮ (ГРУППЕ ЗАБОЛЕВАНИЙ ИЛИ СОСТОЯНИЙ)

1.1. Определение заболевания или состояния (группы заболеваний или состояний).

Ожирение — это гетерогенная группа наследственных и приобретенных заболеваний, связанных с избыточным накоплением жировой ткани в организме [1, 2].

1.2. Этиология и патогенез заболевания или состояния (группы заболеваний или состояний).

Ожирение относится к многофакторным заболеваниям, возникающим в результате определенного взаимодействия генетических и негенетических причин. Роль «наследственности» в развитии ожирения доказывается разной частотой встречаемости данного заболевания в различных этнических группах и более высокой конкордантностью в развитии патологии у однояйцовых близнецов.

Самый частый вид ожирения, связанный с избыточным поступлением калорий в условиях гиподинамии и наследственной предрасположенности, — конституционально-экзогенное (простое, идиопатическое) ожирение.

Генетическая составляющая является определяющей для моногенных и некоторых синдромальных форм ожирения [3]. Значительно реже ожирение в детском и подростковом возрасте связано с применением лекарственных препаратов (например, глюкокортикостероидов, антидепрессантов, нейролептиков (антипсихотиков), противоэпилептических препаратов) или наличием заболеваний и состояний (опухолей гипоталамуса и ствола мозга, лучевой терапией опухолей головного мозга и гемобластозов, травмой черепа, инсультом, гиперкортицизмом, гипотиреозом и другими нейроэндокринными заболеваниями, хромосомными нарушениями).

1.3. Эпидемиология заболевания или состояния (группы заболеваний или состояний).

По данным Всемирной организации здравоохранения (ВОЗ), более миллиарда человек на планете имеют лишний вес, в 2014 г. зарегистрировано более 500 млн больных ожирением. При этом 30 млн детей и подростков Европейского региона имеют избыточную массу тела и 15 млн — ожирение (Health in the European Union. Trends and analysis. ВОЗ, 2009). Одной из самых негативных тенденций можно назвать увеличение числа избыточной массы тела у детей младшего возраста. По оценке ВОЗ, существующие тенденции могут обусловить наличие ожирения у 70 млн детей до 5 лет к 2025 г. [4].

Одно из наиболее крупных популяционных исследований в Российской Федерации, проведенное в 2004 г., включившее 13 700 детей 6–18 лет из 6 регионов (Тверская, Ростовская, Тульская, Брянская, Калужская, Орловская области и остров Сахалин) выявило избыточную массу тела у детей в 5,5–11,8% случаев, а ожирение — у 5,5% детей, проживающих в сельской местности, и 8,5% — в городской [5]. Средний возраст обследуемых составил 13 лет. Исследование 2017–2018 гг., проведенное в г. Москве в рамках программы COSI (Childhood Obesity Surveillance Initiative — инициатива Европейского регионального бюро ВОЗ по эпиднадзору за детским ожирением), включившее 2166 детей 7-летнего возраста, выявило наличие избыточной массы тела у 27% мальчиков и 22% девочек, а ожирение — у 10% и 6% детей соответственно [6].

1.4. Особенности кодирования заболевания или состояния (группы заболеваний или состояний) по Международной статистической классификации болезней и проблем, связанных со здоровьем

1.5. Классификация заболевания или состояния (группы заболеваний или состояний).

Классификация ожирения у детей (В.А. Петеркова, О.В. Васюкова, 2014 [1][2]).

1. По этиологии:

2. По наличию осложнений и коморбидных состояний:

3. По степени ожирения:

1.6. Клиническая картина заболевания или состояния (группы заболеваний или состояний).

Клиническая картина определяется этиопатогенетической формой ожирения. Самая многочисленная группа, на долю которой приходится до 98–99% всех случаев ожирения, — простое (конституционально-экзогенное, идиопатическое) ожирение. Дебют заболевания чаще всего в возрасте после 5 лет или в периоде полового созревания. Как правило, ожирение прогрессирует постепенно, на фоне хороших (часто ускоренных) темпов роста. Наличие стрий, фолликулярного кератоза, полифагии, черного акантоза, артериальной гипертензии и других не всегда коррелирует со степенью ожирения. Характерно наличие избыточной массы тела и ожирения у родственников (родители, бабушки, дедушки). Гипоталамическое ожирение в большинстве случаев отличается быстропрогрессирующим характером, развивается после оперативного вмешательства (лучевой терапии), реже — предшествует периоду постановки диагноза. В случае краниофарингиомы для большинства пациентов характерно замедление темпов роста; для глиом — симптомы преждевременного полового развития; неврологические жалобы (головные боли, нарушение зрения) зависят от локализации и прогрессии опухоли. У детей с гипоталамическим ожирением часто отмечаются нарушения ритма сна и бодрствования и поведенческие нарушения. При моногенных формах ожирение дебютирует в первые месяцы и годы жизни, для большинства синдромальных форм характерна задержка психомоторного развития.

Уровень убедительности рекомендаций С (уровень достоверности доказательств — 5).

Комментарии. Синдромальные формы ожирения характеризуются ранним дебютом ожирения и его быстрым прогрессированием. Для большинства синдромальных форм характерны задержка нервно-психического развития от умеренной до тяжелой степени выраженности, наличие дисморфических признаков и органоспецифических аномалий развития. К настоящему времени изучено не менее 30 синдромов, ассоциированных с ожирением (наиболее известные приведены в табл. 1).

Моногенные формы ожирения (табл. 2) встречаются крайне редко, отличаются ранним дебютом (с первых месяцев жизни до 1 года), полифагией. Для большинства пациентов характерно нормальное нервно-психическое развитие.

**Table:** Таблица 1. Синдромальные формы ожирения

Прадера–Вилли (СПВ)	Болезнь импринтинга, снижение экспрессии генов отцовской аллели 15q11-q13 (SNURF-SNRPN, MRKN3, MAGEL2, NDN, NPAP1).В основном спорадические случаи	1/10 000–1/30 000 новорожденных	С 2 лет жизни	Мышечная гипотония с вялостью сосания, задержка психомоторного развития,низкий рост, акромикрия, лицевой дисморфизм (долихоцефалия, миндалевидный разрез глаз,гипопигментация, страбизм), гипопигментация, гипоплазия наружных половых органов (крипторхизм у мальчиков)	Гипогонадизм смешанного генеза (первичный+вторичный), вторичный гипотиреоз, СТГ-дефицит, вторичный гипокортицизм, апноэ сна (при проведении полисомнографии)
Барде–Бидля(СББ)	BBS1 (11q13)BBS2 (16q12.2)BBS3 (3q11)BBS4 (15q24.1)BBS5 (2q31.1)BBS6 (20p12)BBS7 (4q27)BBS8 (14q31)BBS9 (7p14)BBS10(12q21.2)BBS 11 (9q33.1)BBS12 (4q27)BBS13 (17q23)BBS14(12q21.3)BBS15 (2p15)BBS16 (1q43)BBS17 (3p21)BBS18 (10q25)BBS19 (22q12).Аутосомно-рецессивное	1/135 000–1/175 000 среди европейского населения1/13 500–1/17 500 в изолированных этнических группах и среди населения с высоким процентом близкородственных браков	С первых лет	Низкий рост, задержка умственного развития, прогрессирующая потеря зрения (с 7–8 лет жизни), аномалии развития конечностей (синдактилия, брахидактилия, полидактилия)	Пигментная дистрофия сетчатки, аномалии строения и функции почек, гипогонадизм, нейросенсорная тугоухость
Псевдогипо-паратиреоз 1 А типа	GNAS (20q13).Аутосомно-доминантное наследование	1/100 000–1/300 000 человек популяции	С первых лет	Низкий рост,задержка умственного развития, лунообразное лицо, короткая шея, брахидактилия, укорочение 4 и 5 карпальных и метакарпальных костей, подкожные эктопические оссификаты	Гормональная резистентность:•псевдогипопаратиреоз (высокий уровень паратгормона в крови, гипокальциемия, гиперфосфатемия);•СТГ-дефицит;•гипотиреоз;•гипогонадизм
Ломкой Х-хромосомы	FMR1 (Xq27.3).Х-сцепленное наследование	1/4000–1/8000 человек в популяции	С первых лет	Задержка умственного развития, высокий лоб, выступающий подбородок, большие уши, макроорхидизм, расстройства аутистического спектра	Гипогонадизм
Альстрема	ALMS1 (2p 13-р14).Аутосомно-рецессивное	950 пациентов в мире	С первых лет	Задержка психомоторного развития, нарушение поведения, нистагм, светобоязнь, снижение зрения с 1 года жизни	Нейросенсорная тугоухость, дистрофия сетчатки, сахарный диабет 2 типа, дилатационная кардиомиопатия, нефропатия, нейросенсорная тугоухость, гипогонадизм
Боресона–Форсмана–Лемана	PHF6 (Xq26-27).Х-сцепленное	Около 50 пациентов	С 6–7 лет, умеренное	Гипотония, выраженная задержка умственного развития, низкий рост, микроцефалия, гинекомастия, большие уши	Гипогонадизм, эпилепсия
Паллистера (ульнарно- маммарный синдром)	TBX3 (12q24.21).Аутосомно-доминантное наследование			Дефекты локтевой кости, нарушение развития грудных желез, зубов, задержка полового созревания	
Коэна	COH1 (8q22-q23).Аутосомно-рецессивное	Менее 1000 пациентов	С 8–10 лет, умеренное	Гипотония, сниженный интеллект, выступающие передние резцы, микроцефалия, прогрессирующее снижение зрения, гипермобильность суставов	Пигментная дистрофия сетчатки, нейтропения, гипогонадизм
Карпентера	RAB23	1/1 000 000		«Башенная» форма черепа, синдактилия, полидактилия, гипогонадизм, сниженный интеллект	

**Table:** Таблица 2. Моногенные формы ожирения

Лептина (LEP)	Гомозиготная мутация.Аутосомно-рецессивное	Менее чем у 100 пациентов в мире	Выраженное ожирение с первых дней жизни	Частые респираторные заболевания (иммунодефицит с дефицитом Т-клеточного звена)	Вторичный гипотиреоз, гипогонадотропный гипогонадизм, низкий или неопределяемый уровень лептина (гиполептинемия), гиперинсулинемия
Рецептора лептина (LEPR)	Гомозиготная мутация.Аутосомно-рецессивное	У 2–3% пациентов с ранним ожирением	Выраженное ожирение с первых дней жизни	Частые респираторные заболевания (иммунодефицит с дефицитом Т-клеточного звена)	Вторичный гипотиреоз, гипогонадотропный гипогонадизм, гиперлептинемия, гиперинсулинемия
Проопиомеланокортина (РОМС)	Гомозиготная или компаундная гетерозиготная мутация.Аутосомно-рецессивное	Менее чем у 10 пациентов в мире	Выраженное ожирение с первых месяцев жизни	Гипопигментация (рыжий цвет волос)	Вторичный гипотиреоз, гипогонадотропный гипогонадизм, гипокортицизм, возможен гипогликемический синдром
Прогормонконвертазы 1 типа (PCSK1)	Гомозиготная или компаундная гетерозиготная мутация.Аутосомно-рецессивное или доминантное наследование	Менее чем у 20 пациентов в мире	Выраженное ожирение с первых месяцев жизни	Тяжелая мальабсорбция в неонатальном периоде, полиурия, полидипсия	Вторичный гипотиреоз, гипогонадотропный гипогонадизм, гипокортицизм (повышенные уровни POMC, низкое содержание АКТГ), СТГ-дефицит, постпрандиальная гипогликемия (повышенный уровень проинсулина и низкое содержание инсулина в плазме), центральный несахарный диабет
SIM1	Транслокация хромосомы 1р22.1 и 6q16.2 гена SIM1.Аутосомно-доминантное наследование	Менее чем у 50 пациентов в мире	Выраженное ожирение с первых месяцев жизни	Задержка развития, психоневрологические расстройства (эмоциональная лабильность, расстройства аутистического спектра). Артериальная гипотония. Встречаются признаки СПВ (неонатальная гипотония, дизморфия, низкий рост)	Возможно наличие эндокринопатий. Вторичный гипотиреоз, гипогонадотропный гипогонадизм, СТГ-дефицит
Нейротрофического фактора головного мозга (BDNF) и его рецептора —тирозинкиназы В (NTRK2)	Гетерозиготная мутация de novo.Аутосомно-доминантное наследование	Менее чем у 10 пациентов в мире	Выраженное ожирение с первых месяцев жизни	Задержка моторного и ппсихоречевого развития, гиперактивность, нарушение концентрации внимания и краткосрочной памяти, низкая болевая чувствительность	
Src-подобного адаптерного белка 2(SH2B1)	Гетерозиготная мутация.Аутосомно-доминантное наследование		Выраженное ожирение с первых лет жизни	Низкий конечный рост, задержка психоречевого развития, агрессивное поведение	Выраженная инсулинорезистентность
Киназы супрессора белков Ras 2 типа(KSR2)	Гетерозиготная мутация.Аутосомно-доминантное наследование	Около 65 пациентов в мире	Выраженное ожирение с первых месяцев жизни	Брадикардия	Выраженная инсулинорезистентность, сниженный основной обмен
Tubby-образного белка(TUB)	ГомозиготнаяМутация.Аутосомно-рецессивное наследование	3 сибса	С первых месяцев жизни	Нарушение зрения (снижение остроты),ночная слепота, тугоухость	Пигментная дистрофия сетчатки
Карбоксипептидазы(КПЕ)(CPE)	Гомозиготнаямутация			Задержка умственного развития	Сахарный диабет 2 типа, гипогонадотропный гипогонадизм
Рецептора меланокортина 4-го типа(MC4R)	Гетерозиготная мутация, гомозиготная мутацияАутосомно-доминантное или рецессивное наследование	У 2–6% детей с ожирениемСамая распространенная форма моногенного ожирения!	Выраженное ожирение с первых месяцев жизни	Ускорение темпов роста или высокорослость	Повышенное содержание «тощей массы» и минеральной плотности костей.Гиперинсулинемия.Артериальная гипотония

Нейроэндокринные заболевания являются редкими причинами ожирения в детском возрасте и отличаются характерными клиническими признаками. Так, для гиперкортицизма характерно снижение темпов роста наряду с прогрессирующим ожирением, тогда как появление сухости кожных покровов, непереносимости холода, быстрой утомляемости может свидетельствовать о наличии гипотиреоза.

Общие принципы дифференциальной диагностики различных форм ожирения представлены в приложении (рис. 1).

## 2. ДИАГНОСТИКА ЗАБОЛЕВАНИЯ ИЛИ СОСТОЯНИЯ

Критерии установления диагноза/состояния.

В качестве диагностического критерия избыточной массы тела и ожирения у детей рекомендовано определение величины стандартных отклонений индекса массы тела (SDS ИМТ). С учетом рекомендаций ВОЗ, ожирение у детей и подростков от 0 до 19 лет следует определять как ИМТ, равный или более +2,0 SDS ИМТ, а избыточную массу тела — от +1,0 до +2,0 SDS ИМТ. Нормальная масса тела диагностируется при значениях ИМТ в пределах ± 1,0 SDS ИМТ [1][5][7].

2.1. Жалобы и анамнез.

При сборе анамнеза выявляют вес при рождении, возраст дебюта ожирения, психомоторное развитие, наследственный анамнез по ожирению (включая рост и вес родителей), сахарному диабету 2 типа и сердечно-сосудистым заболеваниям, динамику роста и веса, наличие неврологических жалоб (головные боли, нарушение зрения).

2.2. Физикальное обследование.

Уровень убедительности рекомендаций С (уровень достоверности доказательств — 5).

Уровень убедительности рекомендаций С (уровень достоверности доказательств — 5).

Уровень убедительности рекомендаций С (уровень достоверности доказательств — 5).

Уровень убедительности рекомендаций С (уровень достоверности доказательств — 5).

Уровень убедительности рекомендаций С (уровень достоверности доказательств — 5).

Уровень убедительности рекомендаций С (уровень достоверности доказательств — 5).

2.3. Лабораторные диагностические исследования.

Уровень убедительности рекомендаций С (уровень достоверности доказательств — 5).

Комментарии. Диагноз дислипидемии устанавливается при наличии 2 и более «высоких» и/или «низких» показателей: холестерин ≥5,2 ммоль/л; триглицериды >1,3 ммоль/л (для детей до 10 лет); ≥1,7 (для детей старше 10 лет) ммоль/л; уровень липопротеидов высокой плотности (ЛПВП) ≤0,9 (мальчики) и ≤1,03 (девочки) ммоль/л; уровень липопротеидов низкой плотности (ЛПНП) ≥3,0 ммоль/л. Активность АлАТ, превышающая верхнюю границу нормы, установленной для данной лаборатории, у детей с признаками неалкогольной жировой болезни печени (НАЖБП) по УЗИ при отсутствии других причин синдрома цитолиза (вирусные, метаболические поражения печени и др.) расценивается как проявление стеатогепатита [10]. В сомнительных случаях достоверная диагностика неалкогольного стеатогепатита возможна только после морфологического исследования ткани печени.

Уровень убедительности рекомендаций С (уровень достоверности доказательств — 5).

Комментарии. Нецелесообразно исследование уровня глюкозы в крови с помощью глюкометров для диагностики нарушений углеводного обмена, так как глюкометры не обладают достаточной точностью для убедительной постановки диагноза и могут привести к ошибкам при диагностике [56]. При клинически манифестном сахарном диабете (СД) проводить ОГТТ не рекомендуется, нарушения углеводного обмена можно диагностировать с помощью показателей гликемии натощак, постпрандиально или в течение дня, исследованием уровня гликированного гемоглобина. Проведение ОГТТ детям с ожирением, не достигшим 10 лет, показано при наличии у ребенка дополнительных факторов риска: клинических признаков инсулинорезистентности (акантоз), гестационного диабета у матери, СД у родственников 1-й и 2-й линии родства, при подозрении на врожденные синдромы, связанные с ранним развитием СД и др., и в каждом случае решается индивидуально [57].

2.4. Инструментальные диагностические исследования.

Уровень убедительности рекомендаций С (уровень достоверности доказательств — 5).

Комментарии. УЗИ помогает выявить стеатоз печени и наличие калькулезного холецистита с достаточно высокой точностью. Диагностическими ультразвуковыми признаками жирового гепатоза являются гепатомегалия, неоднородность паренхимы и ослабление ультразвукового сигнала в дистальных отделах печени, обеднение сосудистого рисунка.

Уровень убедительности рекомендаций С (уровень достоверности доказательств — 5).

Комментарии. Ночная полисомнография является золотым стандартом диагностики обструктивного апноэ во сне.

2.5. Иные диагностические исследования

Уровень убедительности рекомендаций С (уровень достоверности доказательств — 5).

Комментарии. Для оценки фактического питания и изменений пищевого статуса используются различные методы, в том числе метод 24-часового (суточного) воспроизведения питания (ведение пищевого дневника) и метод анализа частоты потребления пищи. Метод регистрации потребляемой пищи посредствам пищевого дневника является наиболее точным и достоверным в оценке фактического питания. К его недостаткам можно отнести трудоемкость метода и влияние на привычное питание пациента.

Уровень убедительности рекомендаций С (уровень достоверности доказательств — 5).

Уровень убедительности рекомендаций С (уровень достоверности доказательств — 5).

Уровень убедительности рекомендаций С (уровень достоверности доказательств — 5).

Комментарии. Золотым стандартом диагностики ИР являются эугликемический и гипергликемический клэмп, а также внутривенный глюкозотолерантный тест с частыми заборами крови, оцениваемый с помощью минимальной модели Бергмана [18][19]. К сожалению, эти тесты неприменимы в повседневной практике, так как они весьма продолжительны, дорогостоящи и инвазивны, требуют специально обученного медицинского персонала и сложной статистической обработки результатов. В повседневной практике для оценки ИР при ожирении у детей и подростков наибольшей диагностической значимостью обладают значения стимулированного выброса инсулина и индекса Matsuda, определяемые по данным глюкозотолерантного теста [20]. Значения индекса ниже 2,6 свидетельствуют о наличии ИР [53][60]. К показаниям для проведения глюкозотолерантного теста с оценкой ИР можно отнести наличие у пациента ранее выявленных нарушений углеводного обмена, отягощенный семейный анамнез (по СД 2 типа, гиперандрогении и др.), наличие объективных маркеров ИР — acanthosis nigricans или выраженная гиперпигментация кожных складок шеи, подмышечных или паховой областей, клинические признаки гиперандрогении.

Уровень убедительности рекомендаций С (уровень достоверности доказательств — 4).

Комментарии. Биоимпедансный анализ состава тела основан на измерении электрического сопротивления тканей (импеданса) при прохождении через них низкоинтенсивного электрического тока и позволяет оценить количество жировой и тощей массы, а также воды в организме (композиционный состав тела). Исследование целесообразно для поддержания мотивационной приверженности пациента к лечению, оценки изменения композиционных параметров тела в динамике, но не является обязательным.

Уровень убедительности рекомендаций С (уровень достоверности доказательств — 5).

Комментарии. Основным методом исследования основного обмена в настоящее время является непрямая респираторная калориметрия. Данная методика рекомендована Американской ассоциацией диетологов и нутрициологов и Американской академией педиатрии в качестве предпочтительного метода для оценки основного обмена у детей и подростков. Вместе с тем, учитывая дороговизну метода, необходимость дополнительного обучения врача проведению данного исследования, оценка основного обмена возможна на базе крупных центров с наличием специализированного эндокринологического отделения; не является обязательной.

## 3. ЛЕЧЕНИЕ, ВКЛЮЧАЯ МЕДИКАМЕНТОЗНУЮ И НЕМЕДИКАМЕНТОЗНУЮ ТЕРАПИИ, ДИЕТОТЕРАПИЮ, ОБЕЗБОЛИВАНИЕ, МЕДИЦИНСКИЕ ПОКАЗАНИЯ И ПРОТИВОПОКАЗАНИЯ К ПРИМЕНЕНИЮ МЕТОДОВ ЛЕЧЕНИЯ

Изменение образа жизни (диетотерапия, расширение физической активности и коррекция пищевого поведения) у детей и подростков с ожирением или избыточной массой тела, а также членов их семьи составляет основу терапии ожирения и его профилактики. В случае неэффективности модификации образа жизни возможно использование фармакологических средств, список которых у детей и подростков на сегодняшний день ограничен орлистатом и лираглутидом. Бариатрическая хирургия является еще одним методом лечения морбидного осложненного ожирения у подростков.

Целью лечения ожирения у детей и подростков является в краткосрочном периоде удержание значения SDS ИМТ (в течение 6–12 мес наблюдения), в долгосрочном периоде — уменьшение величины SDS ИМТ, достижение «избыточной массы тела» и «нормальной массы тела», нормальное физическое и соматическое развитие ребенка, развитие самостоятельности и мотивации к самоконтролю пищевого поведения, профилактика ассоциированных с ожирением коморбидных состояний.

3.1. Диетотерапия.

Уровень убедительности рекомендаций А (уровень достоверности доказательств — 1).

Комментарии. Современный тренд в диетологии детского ожирения — нормокалорийный рацион по возрасту с достаточным количеством белков, углеводов, витаминов и микроэлементов и необходимым минимумом жиров, составленный с учетом вкусовых предпочтений ребенка. Все виды диет — гипокалорийная, кетогенная, низкожировая, со сниженным гликемическим индексом и другие являются альтернативными вариантами терапии, применяются по показаниям и часто в условиях специализированных отделений.

3.2. Физические нагрузки и профилактика «малоподвижного образа жизни».

Уровень убедительности рекомендаций С (уровень достоверности доказательств — 5).

Уровень убедительности рекомендаций С (уровень достоверности доказательств — 5).

Комментарии. Согласно глобальным рекомендациям ВОЗ, адекватная физическая активность для детей и подростков в возрасте 6–17 лет подразумевает ежедневные занятия продолжительностью не менее 60 минут в день. Физическая активность свыше 60 минут в день дает дополнительные преимущества для здоровья. Рекомендованная ежедневная продолжительность физических нагрузок (60 минут и более) может складываться в течение дня из более коротких нагрузок (например, 2 раза в день по 30 минут). Минимально эффективными считаются 10-минутные периоды физической активности — от умеренной до высокой интенсивности.

Уровень убедительности рекомендаций С (уровень достоверности доказательств — 5).

Комментарии. Согласно рекомендациям ВОЗ от 2019 г. по вопросам физической активности, сна и малоподвижного образа жизни у детей до 5 лет, разработаны отдельные дефиниции по продолжительности физической активности для различных возрастных групп. Под физической активностью для детей младшего возраста подразумеваются различные игры: например, лежа на полу, с игрушками, ползание, гимнастика для малышей и т.д. Если ребенок еще не может ползать, рекомендуется проводить не меньше 30 минут в день лежа на животе.

Уровень убедительности рекомендаций С (уровень достоверности доказательств — 5).

Уровень убедительности рекомендаций С (уровень достоверности доказательств — 5).

Уровень убедительности рекомендаций С (уровень достоверности доказательств — 5).

Комментарии. Рекомендации ВОЗ у детей до 5 лет лимитируют время, проводимое маленьким ребенком в удерживающих устройствах и перед экраном смартфона или телевизора (например, просмотр мультфильмов по телевизору или на переносных устройствах, игры на смартфонах и др.). Время, в течение которого ребенок находится в удерживающих устройствах, исключая сон, родителям стоит использовать для общения: читать сказки, рассказывать стихи, петь песенки. Под удерживающими устройствами имеются в виду различные люльки, коляски, детские кресла или переноски.

Кроме того, рекомендации ВОЗ 2019 г. определяют продолжительность сна у детей до 4 лет. Так, для детей до года длительность сна составляет 14–17 ч (в возрасте от 0 до 3 мес) или 12–16 ч (в возрасте от 4 до 11 мес), включая дневной сон. Для детей от 1 года до 2 лет длительность сна составляет 11–14 ч, включая дневной сон. Для детей от 3 до 4 лет длительность сна составляет 10–13 ч. Во всех возрастных группах важно соблюдение режима дня [25].

Уровень убедительности рекомендаций С (уровень достоверности доказательств — 5).

Комментарии. Необходимо сокращение времени, затрачиваемого на физически неактивные виды времяпрепровождения: телевидение, видеофильмы, компьютерные игры, «брожение» по Интернету. С первого дня и на протяжении всего 1-го месяца экранное время сокращается на 30 минут, со 2-го месяца — на 45 минут, с 3-го месяца — на 60 минут и т.д.

3.3. Медикаментозная терапия.

Уровень убедительности рекомендаций А (уровень достоверности доказательств — 1).

Комментарии. Медикаментозная терапия ожирения у подростков ограничена. Препараты, разрешенные для лечения ожирения у детей старше 12 лет в мире и Российской Федерации, — это лираглутид и орлистат.

Уровень убедительности рекомендаций В (уровень достоверности доказательств — 2).

Комментарии. Лираглутид является аналогом глюкагоноподобного пептида 1 (ГПП-1). На уровне гипоталамуса лираглутид, активируя рецепторы ГПП-1, усиливает сигналы насыщения и ослабляет сигналы голода, тем самым сокращая потребление пищи. Кроме того, лираглутид глюкозозависимым путем стимулирует секрецию инсулина и уменьшает секрецию глюкагона. Эффективность и безопасность лираглутида у подростков с ожирением в возрасте 12–17 лет оценена в рандомизированном клиническом исследовании SCALЕ TEENS. Лираглутид снижал массу тела (в среднем на 2,7 кг по сравнению с набором веса в группе плацебо +2,1 кг), уменьшал величину SDS ИМТ больше (на 0,25), чем плацебо (0,02); также отмечено большее достижение пациентами (5 и 10%) снижения массы тела в группе лираглутида по сравнению с плацебо [40][41].

Препарат вводится подкожно 1 раз в сутки в любое время, независимо от приема пищи, в область живота, бедра или плеча. Начальная доза составляет 0,6 мг в сутки с последующей стандартной титрацией дозы препарата согласно инструкции: доза увеличивается на 0,6 мг с интервалами не менее 1 нед. Дозу препарата следует увеличивать до тех пор, пока не будет достигнуто значение 3,0 мг (терапевтическая доза) или максимально переносимая доза. Среди побочных действий описаны диспепсические явления (снижение аппетита, тошнота, рвота, запор, диарея), которые отмечаются, как правило, в первые недели лечения, в большинстве случаев носят преходящий характер и не требуют отмены терапии.

Уровень убедительности рекомендаций В (уровень достоверности доказательств — 2).

Комментарии. Орлистат является ингибитором желудочной и панкреатической липаз, которые участвуют в гидролизе триглицеридов и необходимы для всасывания жиров в тонком кишечнике. В результате действия препарата нарушается расщепление пищевых жиров и уменьшается их всасывание. После отмены препарата его действие быстро прекращается, а активность липаз восстанавливается. Эффективность орлистата в комплексной терапии ожирения у подростков оценена в контролируемых клинических исследованиях. Согласно данным работам, средняя динамика веса в группе орлистата составила от +0,53 кг (12 мес терапии, 12 мес наблюдения, 539 подростков) [30], до –6,9 кг (6 мес терапии, 60 пациентов) [31]. Орлистат назначается по 1 капсуле (120 мг) перед основными приемами пищи, максимальная суточная доза составляет 360 мг (3 капсулы, по 1 капсуле 3 раза в день). Длительность лечения может составлять от 3 до 12 мес.

Уровень убедительности рекомендаций С (уровень достоверности доказательств — 5).

Комментарии. Применение метформина в педиатрической практике разрешено в возрастной группе старше 10 лет с установленным диагнозом СД 2 типа [32–35]. Метаанализы демонстрируют умеренный положительный эффект метформина, выражающийся преимущественно в стабилизации веса и SDS ИМТ, а также улучшении метаболического профиля, инсулинорезистентности у детей и подростков с ожирением [34][36]. Согласно данным, представленным Международным консорциумом детских эндокринологов по вопросам диагностики и лечения синдрома поликистозных яичников (СПЯ) у подростков, метформин оказывает благоприятное влияние на течение СПЯ у пациенток с избыточной массой тела и ожирением в краткосрочном периоде (6 мес). Также терапия метформином снижает частоту ановуляции и уровень тестостерона у пациенток с СПЯ без ожирения. [37]. Таким образом, терапия метформином «офф-лейбл» наиболее целесообразна в группе пациенток с гиперандрогенией и нарушениями менструального цикла, входящих в группу риска развития СПЯ.

Уровень убедительности рекомендаций С (уровень достоверности доказательств — 5).

Уровень убедительности рекомендаций В (уровень достоверности доказательств — 2).

Комментарии. Согласно данным многочисленных исследований, терапия соматропином, особенно при назначении в раннем возрасте (до развития ожирения), приводит к улучшению антропометрических параметров и композиционного состава тела (снижению жировой и увеличению мышечной массы), что может способствовать уменьшению риска развития ожирения у этих пациентов (при условии соблюдения диеты и режима двигательной активности) [66]. Перед началом и на фоне терапии соматропином проводится оценка антропометрических параметров, состояния аденотонзиллярной системы, показателей костного возраста, значений уровня инсулиноподобного фактора роста 1 (ИФР-1), параметров углеводного обмена (исследования уровней глюкозы, иммунореактивного инсулина, гликированного гемоглобина). Учитывая потенциальный эффект соматропина на гипертрофию лимфоидной ткани носоглоточного кольца и ухудшение параметров дыхания во сне с риском развития апноэ, рекомендовано проведение полисомнографии как перед, так и на фоне терапии, особенно в первые 3–6 мес лечения соматропином. Средняя суточная доза соматропина для лечения СПВ — 1 мг/м2/сут, однако начинать терапию, особенно детям раннего возраста, рекомендовано с меньших доз (0,5 мг/м2/сут) с последующей титрацией до среднесуточной под контролем значений ИФР-1, избегая превышения референсных значений [38]. У детей с СПВ раннего возраста (до 2 лет жизни) терапия соматропином в меньших дозах (0,6 мг/м2/сут) показала эффективность, аналогичную таковой при использовании среднетерапевтических доз (1 мг/м2/сут), и характеризовалась меньшим количеством побочных эффектов на начальном этапе лечения [67]. Противопоказаниями для назначения соматропина у пациентов с СПВ являются тяжелое осложненное ожирение, некомпенсированный СД, тяжелая степень апноэ, активные злокачественные новообразования, психотические расстройства [38][39][66].

3.4. Хирургическое лечение.

- ИМТ >35 кг/м² в сочетании с тяжелыми осложнениями (неалкогольный стеатогепатит, сахарный диабет 2 типа, синдром обструктивного апноэ во сне, болезнь Блаунта, тяжелая артериальная гипертензия).

- ИМТ >40 кг/м² (SDS ИМТ > 4,0 для данного пола и возраста) независимо от наличия осложнений.

- Завершенное или близкое к завершению физическое развитие (частичное или полное закрытие зон роста), достижение 4–5 стадий полового развития по шкале Таннера.

- Документально подтвержденная неэффективность консервативных методов лечения ожирения в течение 12 мес в специализированных центрах.

- Отсутствие психических заболеваний и расстройств пищевого поведения (в том числе обусловленных наличием синдромальных и гипоталамических форм ожирения).

- Готовность/способность подростка и членов его семьи к длительному и регулярному послеоперационному динамическому наблюдению.

Уровень убедительности рекомендаций С (уровень достоверности доказательств — 5).

Комментарии. Хирургические методы лечения морбидного ожирения (бариатрическая хирургия) у подростков получают все большее распространение в мире в последние десятилетия. Основными преимуществами бариатрической хирургии являются быстрое снижение веса [47–51], улучшение метаболических показателей и качества жизни пациентов с морбидным ожирением [50–52]. Всем подросткам после бариатрических операций требуется мониторинг уровня витаминов и микроэлементов для своевременной диагностики их дефицита. Наиболее часто у данной группы пациентов развивается дефицит кальция и витамина D, которые при несвоевременной коррекции приводят к развитию вторичного гиперпаратиреоза и остеопороза. Часто регистрируется дефицит железа, фолиевой кислоты и других витаминов (тиамин, пиридоксин, цианокобаламин), а также жирорастворимых витаминов [50–52]. Тем не менее частое развитие стойкого дефицита витаминов и микроэлементов, высокий процент повторных оперативных вмешательств, необходимость наблюдения мультидисциплинарной командой специалистов и низкая комплаентность больных ограничивают широкое применение метаболической хирургии в лечении морбидного ожирения у подростков.

## 4. МЕДИЦИНСКАЯ РЕАБИЛИТАЦИЯ И САНАТОРНО-КУРОРТНОЕ ЛЕЧЕНИЕ, МЕДИЦИНСКИЕ ПОКАЗАНИЯ И ПРОТИВОПОКАЗАНИЯ К ПРИМЕНЕНИЮ МЕТОДОВ МЕДИЦИНСКОЙ РЕАБИЛИТАЦИИ, В ТОМ ЧИСЛЕ ОСНОВАННЫХ НА ИСПОЛЬЗОВАНИИ ПРИРОДНЫХ ЛЕЧЕБНЫХ ФАКТОРОВ

Специфические реабилитационные мероприятия не предусмотрены.

## 5. ПРОФИЛАКТИКА И ДИСПАНСЕРНОЕ НАБЛЮДЕНИЕ, МЕДИЦИНСКИЕ ПОКАЗАНИЯ И ПРОТИВОПОКАЗАНИЯ К ПРИМЕНЕНИЮ МЕТОДОВ ПРОФИЛАКТИКИ

Уровень убедительности рекомендаций С (уровень достоверности доказательств — 5).

Комментарии. Врач-педиатр наблюдает детей дошкольного и младшего школьного возраста с избыточной массой тела или метаболически неосложненным ожирением. Дети до года наблюдаются врачом-педиатром ежемесячно. Им проводят контроль антропометрических показателей, SDS ИМТ. При развитии ожирения показана консультация детского эндокринолога.

Дети раннего возраста (1–3 года) наблюдаются врачом-педиатром 1 раз в 3–6 мес. При развитии ожирения также показана консультация детского эндокринолога, а при сопутствующей задержке психомоторного развития — консультация генетика.

Диспансерное наблюдение детей дошкольного и младшего школьного возраста с избыточной массой тела может проводиться как врачом-педиатром, так и врачом детским эндокринологом. Самым важным компонентом такого наблюдения по-прежнему будет оценка динамики антропометрических показателей, SDS ИМТ, оценка образа жизни ребенка и подробная беседа с родителями. В первые 3 мес необходимы ежемесячные визиты, далее (при положительной динамике) — 1 раз в 6–12 мес.

При диспансерном наблюдении подростков с ожирением показана консультация врача детского эндокринолога. Обязательным является скрининг коморбидных состояний. Диспансерное наблюдение осуществляется ежемесячно в первые 3 мес, далее (при положительной динамике) — 1 раз в 6 мес. Дети с осложненным ожирением наблюдаются у врача детского эндокринолога 1 раз в 3–6 мес. При сохранении осложнений объем обследований и наблюдений узкими специалистами определяется индивидуально.

При нормализации массы тела дети наблюдаются у врача-педиатра в декретированные сроки для здоровых детей (I группа здоровья).

Уровень убедительности рекомендаций С (уровень достоверности доказательств — 5).

## 6. ОРГАНИЗАЦИЯ ОКАЗАНИЯ МЕДИЦИНСКОЙ ПОМОЩИ

Показания для госпитализации в медицинскую организацию.

1. Форма — плановая; условия — стационар, дневной стационар:

a.комплексный скрининг осложнений, в отсутствие возможности обследования в амбулаторных условиях;

b.комплексное лечение с проведением Школы для пациентов с избыточной массой тела и ожирением (дневной стационар), в отсутствие возможности лечения и проведения Школы в амбулаторных условиях;

c.при планировании хирургического лечения ожирения.

2. Форма — экстренная, неотложная; условия — стационар: не предусмотрены. При развитии неотложных состояний, связанных с ухудшением течения коморбидных заболеваний (гипертонический криз, острый живот при ЖКБ, апноэ), показана госпитализация пациента в профильное отделение согласно основному неотложному состоянию.

Показания к выписке пациента из медицинской организации.

1. Форма — плановая; условия — стационар, дневной стационар:

a.проведение запланированного обследования/лечения.

2. Форма — экстренная, неотложная; условия — стационар: купирование жизнеугрожающего состояния.

## ДОПОЛНИТЕЛЬНАЯ ИНФОРМАЦИЯ

Источники финансирования. Работа выполнена по инициативе авторов без привлечения финансирования.

Конфликт интересов. Авторы декларируют отсутствие явных и потенциальных конфликтов интересов, связанных с содержанием и публикацией настоящей статьи.

Участие авторов. Все авторы одобрили финальную версию статьи перед публикацией, выразили согласие нести ответственность за все аспекты работы, подразумевающую надлежащее изучение и решение вопросов, связанных с точностью или добросовестностью любой части работы.

## ПРИЛОЖЕНИЕ

**Table:** Таблица 3. Критерии оценки качества медицинской помощи

№	Критерии качества	Уровень убедительности рекомендаций	Уровень достоверности доказательств
1	Выполнено измерение роста и веса с оценкой SDS ИМТ	С	5
2	Выполнено измерение артериального давления	С	5
3	Выполнена клиническая оценка полового развития на основании шкалы Таннера	GPP	GPP
4	Выполнена оценка состояния кожных покровов, наличия и характера стрий, акантоза, андрогензависимой дермопатии	GPP	GPP
5	Выполнен клинический скринингсиндромальных/моногенных форм ожирения	С	5
6	Выполнен биохимический анализ крови по оценке нарушений липидного обмена, включающий исследование уровней общего холестерина, ЛПНП, ЛПВП, триглицеридов	С	5
7	Выполнено определение активности АлАТ и АсАТ в крови	С	5
8	Проведено УЗИ органов брюшной полости (комплексного)	С	5
9	Проведена оценка состояния углеводного обмена (исследование уровня глюкозы крови натощак /проведение глюкозотолерантного теста)	С	5
10	При подозрении на гипоталамическое ожирение проведена МРТ головного мозга	С	5
11	Проведена оценка фактического питания с использованием 24-часового (суточного) воспроизведения питания с помощью пищевого дневника	С	5
12	Выполнена консультация пациента и/или родителя по вопросам рационализации питания и физической активности	С	5

**Figure:**
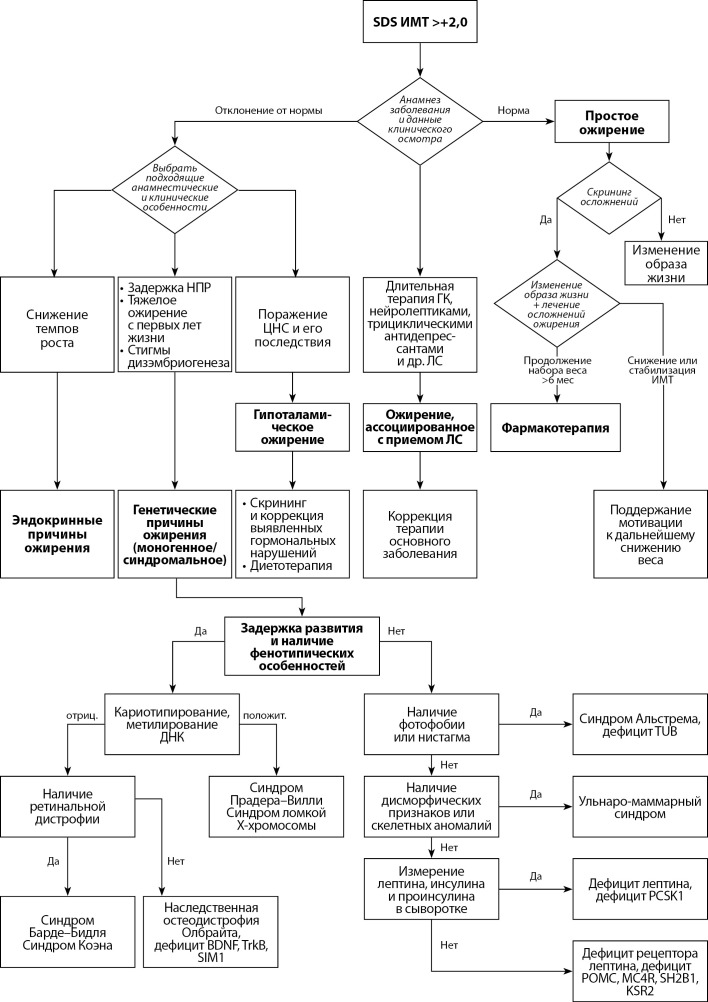
Рисунок 1. Алгоритм действий врача для диагностики и лечения ожирения у детей
